# A Novel Therapeutic Target for Small-Cell Lung Cancer: Tumor-Associated Repair-like Schwann Cells

**DOI:** 10.3390/cancers14246132

**Published:** 2022-12-12

**Authors:** Shuhui Cao, Yue Wang, Yan Zhou, Yao Zhang, Xuxinyi Ling, Lincheng Zhang, Jingwen Li, Yu Yang, Weimin Wang, Michael R. Shurin, Hua Zhong

**Affiliations:** 1 Department of Pulmonary, Shanghai Chest Hospital, School of Medicine, Shanghai Jiao Tong University, Shanghai 200030, China; 2 Department of Pathology, University of Pittsburgh Medical Center, Pittsburgh, PA 15261, USA; 3 Department of Immunology, University of Pittsburgh Medical Center, Pittsburgh, PA 15261, USA

**Keywords:** small-cell lung cancer, Schwann cells, tumor progression, miRNA, gene expression

## Abstract

**Simple Summary:**

Schwann cells (SCs) have been reported to support tumor spreading and metastasis formation in both the in vitro and in vivo models. However, the role of SCs in the progression of small-cell lung cancer (SCLC) has not been investigated. This study demonstrated the crosstalk between SCLC and tumor-associated SCs (TA-SCs) and constructed the mRNA-miRNA-lncRNA network that may regulate tumor cell-SC interaction and cancer progression.

**Abstract:**

Small-cell lung cancer (SCLC), representing 15–20% of all lung cancers, is an aggressive malignancy with a distinct natural history, poor prognosis, and limited treatment options. We have previously identified Schwann cells (SCs), the main glial cells of the peripheral nervous system, in tumor tissues and demonstrated that they may support tumor spreading and metastasis formation in the in vitro and in vivo models. However, the role of SCs in the progression of SCLC has not been investigated. To clarify this issue, the cell proliferation assay, the annexin V apoptosis assay, and the transwell migration and invasion assay were conducted to elucidate the roles in SCLC of tumor-associated SCs (TA-SCs) in the proliferation, apoptosis, migration, and invasion of SCLC cells in vitro, compared to control group. In addition, the animal models to assess SC action’s effects on SCLC in vivo were also developed. The result confirmed that TA-SCs have a well-established and significant role in facilitating SCLC cell cancer migration and invasion of SCLC in vitro, and we also observed that SC promotes tumor growth of SCLC in vivo and that TA-SCs exhibited an advantage and show a repair-like phenotype, which allowed defining them as tumor-associated repair SCs (TAR-SCs). Potential molecular mechanisms of pro-tumorigenic activity of TAR-SCs were investigated by the screening of differentially expressed genes and constructing networks of messenger-, micro-, and long- non-coding RNA (mRNA-miRNA-lncRNA) using DMS114 cells, a human SCLC, stimulated with media from DMS114-activated SCs, non-stimulated SCs, and appropriate controls. This study improves our understanding of how SCs, especially tumor-activated SCs, may promote SCLC progression. Our results highlight a new functional phenotype of SCs in cancer and bring new insights into the characterization of the nervous system-tumor crosstalk.

## 1. Introduction

Lung cancer (LC) is a major cause of morbidity and mortality worldwide [1,2]. In the era of personalized medicine, the tumor microenvironment is an important area of basic and clinical research for further improvement of individualized cancer diagnosis, prognosis, and treatment [3]. The tumor microenvironment consists of cancer stem cells, malignant cells, fibroblasts, immune cells, endothelial cells, pericytes, and adipose cells, as well as various biomolecules supplied by the tumor stromal elements, infiltrating cells, and newly formed or extended capillaries [4]. Different elements of the peripheral nervous system (PNS) have been identified within and around solid tumors [5], and their involvement in supporting tumor growth, spreading, and formation of metastasis has been demonstrated [6,7,8,9]. However, the clinical significance of different PNS cells in controlling the LC microenvironment has not yet been evaluated.

Schwann cells (SCs), primary glial cells of the PNS that are vital in the maintenance and regeneration of neurons [10], have been recently recognized as important players in the tumor microenvironment [11,12]. For instance, in melanoma, SCs support tumor growth via different mechanisms [13]. Interestingly, tumor-associated SCs (TA-SCs) functionally and phenotypically resemble the so-called repair SCs described in Wallerian degeneration—a process orchestrated by SCs after PNS injury or trauma prior to neuronal axon regeneration [13,14]. Transcriptome profiling confirmed these conclusions, showing that genes associated with SC dedifferentiation were overexpressed in lung cancer samples, while genes associated with SC migration inhibition were down-regulated [15]. Deborde et al. found that SCs promote perineural invasion (PNI)—a route of metastatic spread in pancreatic cancer through direct contact with cancerous cells in a process dependent on SC expression of NCAM1 [16]. Although PNI is common for spreading pancreatic, prostate, colon, gastric, and head and neck carcinomas, it is not a common way of invasion in lung cancer. However, our previous results have revealed SCs in lung cancer tissue and demonstrated that SCs could, directly and indirectly, interact with lung cancer cell lines stimulating their proliferation, transmigration, and formation of metastasis in vivo [17,18].

Small-cell lung cancer (SCLC) is an aggressive neuroendocrine subtype of lung cancer, accounting for approximately 15% of lung cancers and having a distinct natural history and poor prognosis [19]. Given its propensity to spread early, patients are often diagnosed with metastatic disease. Although immunotherapy is an emerging treatment and chemotherapy combined with immunotherapy has modestly improved overall survival, SCLC remains a type of lung cancer with a very poor prognosis [20,21]. Brain metastases are common in SCLC patients, where 10–25% presenting with brain metastases at diagnosis and 40–60% developing brain metastases overall [22]. Furthermore, association with Paraneoplastic Neurological Syndrome is significantly higher in SCLC than in other solid malignancies [23]. However, nothing is known about interactions between neurons or neuroglial SCs and SCLC cells and whether SCs may support SCLC invasion and metastasis.

The goal of this study was to characterize the interaction of human SCs with SCLC cells and determine the molecular mechanisms of this new phenomenon. We revealed that SCs have a significant role in facilitating SCLC cell migration and invasion in vitro. We also observed that SCs, especially TA-SCs, strongly promoted the growth of SCLC in vivo primary molecular mechanisms of SCLC, and cell-SC crosstalk was also established.

## 2. Materials and Methods

### 2.1. Cell Cultures

Primary human SCs isolated from spinal nerves were purchased from ScienCell and cultured in an SC medium (Cat#SC-1701, ScienCell Research Laboratories, Carlsbad, CA, USA). Human DMS114, DMS53, and H1048 SCLC cell lines were purchased from the American Type Culture Collection (ATCC, Manassas, VA, USA). All SCLC cells were maintained in complete RPMI-1640 medium (Cat#30809.01, HyClone Laboratories, Inc., South Logan, UT, USA) supplemented with 0.1 mM non-essential amino acids, 10% heat-inactivated fetal bovine serum (Cat#10099141C, FBS; Gibco-Invitrogen Corporation, Carlsbad, CA, USA), 2 mM glutamine, 100 U/mL penicillin, and 100 μg/mL streptomycin.

### 2.2. Cell-Conditioned Media

Different cell-conditioned media were used in the experiments. The SCLC-conditioned medium was obtained from SCLC cells first cultured in the RPMI-1640 medium for 24 h followed by culture in the complete RPMI-1640 medium supplemented with 2% FCS for 48 h that reached the number of cells about 3 × 10^6^ (DMS114), 4 × 10^6^(DMS53), and 8 × 10^6^ (H1048) in a 10 cm dish. The TA-SC-conditioned medium was obtained from SCs that were initially activated by 10% (*v/v*) of the SCLC-conditioned medium for 48 h and then cultured in the RPMI-1640 medium supplemented with 2% FCS for 48 h, reaching the number of cells about 3 × 10^5^ in a T75 flask. Conditioned media from TA-SC cultures include cell-free supernatants from the SCs activated by different tumor cell lines: SC/DMS114-con, SC/DMS53-con, and SC/H1048-con. The control SC-conditioned medium was collected from SCs generated in the SC medium and when 1 × 10^5^ cells were re-cultured in an RPMI-1640 medium supplemented with 2% FCS for 48 h, reaching cell number about 3 × 10^5^. All conditioned media were stored at −80 °C.

### 2.3. Xenograft Experiments

All animal protocols were approved by the Institutional Animal Care and Use Committee of the Model Animal Research Institute of Shanghai Jiao Tong University (Shanghai, China) and conducted in the Model Animal Research Center of Shanghai Chest Hospital (Shanghai, China). Six-week-old female immunodeficient (nu/nu) mice were obtained from the Model Animal Research Center of Shanghai Jiao Tong University. Mice were subcutaneously administered with 250 μL PBS containing (i) DMS114 cells (3 × 10^6^) together with the control SCs (3 × 10^6^), (ii) DMS114 cells (3 × 10^6^) together with SCs (3 × 10^6^) pretreated with minocycline (40 μM/48 h), or (iii) DMS114 cells (3 × 10^6^) alone. All mice were analyzed 40 days after the treatment. Tumor volume was calculated with the formula Volume = π/6 × Length × Width × Hight. All animal experiments included at least six mice per group and were reproduced twice.

### 2.4. Cell Migration Assay

The effects of SCs on the migration of DMS114, DMS53, and H1048 cells were determined using the transwell migration assay (pore size 8 μm; Cat#3422, Corning, Inc., New York, NY, USA). Briefly, tumor cells (10,000/insert) resuspended in 200 μL of a complete RPMI-1640 medium supplemented with 2% FCS were added to the upper chamber, and 750 μL of the TA-SC-conditioned medium (SC/DMS114-con med, SC/DMS53-con med, and SC/H1048-con med), the control SC-conditioned medium, and the RPMI-1640 medium supplemented with 2% FCS (medium) were added to the lower chamber of the device. After 20 h, the non-migrating cells adhered to the upper chamber were carefully removed with a cotton swab, and the cells that migrated to the underside of the membrane in nine different fields were fixed, stained with a Diff-Quick Stain Kit (Cat#40748ES60, Yeasen Biotechnology, Inc., Shanghai, China), and counted on a grid in ten high-power fields. Each group of cells was tested in duplicates and all experiments were repeated at least three times.

### 2.5. Cell Invasion Assay

For the invasion assay, polycarbonate membranes (pore size 8 μm; Cat#3422, Corning, Inc., New York, NY, USA) were precoated with a growth factor reduced Matrigel (1.0 mg/mL; Cat#356234, BD Biosciences, San Jose, CA, USA). Specifically, 30–50 μL of Matrigel was added to a 24-well Transwell insert and solidified for 30 min at 37 °C to form a thin gel layer. Cell loading and counting were carried out as described for the migration assay above. Each group of cells was tested in duplicates and all experiments were repeated at least three times.

### 2.6. Cell Proliferation Assay

The cell proliferation rate was determined using the Cell Counting Kit-8 (CCK-8; Cat#C0038, Biyuntian Biotechnology Co., Ltd., Shanghai, China) in accordance with the manufacturer’s protocol. Briefly, SCLC cells were seeded into the wells of 96-well plates at a density of 5000 cells per well. Cells were cultured in the TA-SCs medium (SC/DMS114-con med, SC/DMS53-con med, and SC/H1048-con med; 10% *v/v*), the SCs medium (SC-con med, 10% *v/v*), and the RPMI-1640 medium supplemented with 2% FCS (medium, 10% *v/v*). After 24 h, 10 μL of CCK-8 solution was added to all wells and the cells were incubated for 2 h. The resulting color was evaluated at a wavelength of 450 nm using iMark™ Microplate Absorbance Reader (Bio-Rad Laboratories, Hercules, CA, USA). Each assay was performed in triplicates and repeated two times.

### 2.7. Apoptosis Assay

The proportion of apoptotic cells was quantified using Annexin V-fluorescein isothiocyanate (FITC)/propidium iodide (PI) Apoptosis Detection Kit (Cat# 40302ES20, Yeasen Biotechnology Co., Ltd., Shanghai, China). Tumor cells were cultured in the TA-SC-conditioned medium (SC/DMS114-con med, SC/DMS53-con med, and SC/H1048-con med,10% *v/v*), the SC-conditioned medium (SC-con med, 10% *v/v*), and the RPMI-1640 medium supplemented with 2% FCS (medium, 10% *v/v*), washed once with phosphate-buffered saline, resuspended in 200 μL of binding buffer with 5 μL of Annexin V-FITC and 10 μL of PI, and incubated in the dark at room temperature for 15 min. Then, 400 μL of binding buffer was added to each sample and the cells were immediately analyzed using a BD^®^ LSR II Flow Cytometer with FACSDiva software (BD Biosciences). Cells were first gated on forward scatter (FSC−) versus side scatter (SSC−) characteristics before excluding doublets using consecutive gating (FSC-Area versus FSC-Width and SSC-Area versus SSC-Width plots). The populations of Annexin V+/PI−, Annexin V+/PI+, Annexin V−/PI+, and Annexin V−/PI− cells were then calculated with quadrant gates. Data were analyzed using FlowJo v 9.6.3 software (BD Biosciences, San Jose, CA, USA).

### 2.8. RNA Extraction

Total RNA was isolated from DMS114 cells cultured with the SC/DMS114-conditioned medium (10% *v/v*), the SC-conditioned medium (10% *v/v*), and the RPMI 1640 medium supplemented with 2% FCS (10% *v/v*) using TRIzol reagent (Cat#15596026, Thermo Fisher Scientific Corporation, Inc, Carlsbad, CA, USA.) in accordance with the manufacturer’s instructions.

### 2.9. RNA Sequencing

High-throughput RNA sequencing of multiple samples was performed using a HiSeq Sequencing System (Illumina, Inc., San Diego, CA, USA). According to the distribution characteristics of the low-quality scores of the Illumina sequencing data concentrated at the end. Trim Galore software (https://www.bioinformatics.babraham.ac.uk/projects/trim_galore/, accessed on 1 July 2022) was used to dynamically remove the linker sequence fragments and low-quality fragments from the 3’ ends of the sequencing data. Quality control analysis on the preprocessed data was performed using FastQC software (http://www.bioinformatics.babraham.ac.uk/projects/fastqc/, accessed on 1 July 2022).

### 2.10. Quality Control

The G+C content of each read was calculated, and the genome results were analyzed based on comparisons of the reads to evaluate the nucleic acid composition. Repeated evaluations of the reads were based on the genome results of the read comparisons. Next, for each sample, STAR software (Star Software Technologies, Amritsar, India) was used to compare the preprocessed sequences with the reference genome sequence of the sequenced species, and then to check whether the sequencing reads are uniform and whether there is a 5′/3′ deviation. Analysis of variable splicing site annotations was based on comparisons of the annotation information of the reference genome with the known gene model for each sample, and the known splice junctions were compared to obtain the number and proportion of new splice junctions in the current transcriptome. Evaluation of variable shear saturation was performed to determine whether the sample sequencing depth agreed with the variable shear site.

### 2.11. Identification of Differentially Expressed Genes (DEGs)

The general-purpose read summarization function “featureCounts” was used to obtain the count matrix of DEGs. According to the experimental design of the sample, DESeq2 (https://bioconductor.org/packages/release/bioc/html/DESeq2.html, accessed on 1 July 2022) was used to identify DEGs among different sample groups [24]. An adjusted probability (*p*) value ≤ 0.05 and greater than or equal to two times the differential expression range were set at parameters to screen DEGs between two groups. Two-way hierarchical clustering was used to screen DEGs between sample groups. The results are displayed as a heat map. The clustering parameters included the Pearson correlation as the distance metric and the average linkage as the linkage rule.

### 2.12. Gene Ontology (GO) Annotations and the Kyoto Encyclopedia of Genes and Genomes (KEGG) Pathway Analyses

All genes/transcripts were used as the background list and the DEGs/transcripts as the candidate list filtered from the background list. The hypergeometric distribution test was used to calculate the probability of whether the GO or KEGG feature set is significantly enriched in the DEG/transcript list. The *p*-value was corrected with the Benjamini–Hochberg procedure to obtain the false discovery rate.

### 2.13. ceRNA Network Analysis

Predictive analysis was conducted using Miranda software (https://cbio.mskcc.org/miRNA2003/miranda.html, accessed on 1 July 2022) with the parameters Tot_Energy ≤ −20 and Total_Score ≥ 180 [25]. The public databases Targetscan, miRDB, mirTarBase, and miRwalk were used to further verify that the micro-RNA (miRNA) target genes met the intersection of Miranda software and the prediction results of at least one public database [26,27,28,29]. The intersection with the lncBaseV2 database and the resulting lncRNA was adopted as the miRNA target genes. A ceRNA network was constructed based on the targeting relationship between the miRNA and genes and the expression trends of lncRNA and mRNA.

### 2.14. Statistical Analysis

Results are expressed as the mean ± SEM. Two-group analyses were performed using an unpaired *t*-test. Three or more groups with one independent variable were analyzed using a one-way ANOVA test. Three or more groups with two independent variables were analyzed using a two-way ANOVA test. Analyses were performed using the Prism (GraphPad Software, San Diego, CA, USA) and SigmaStat (SyStat Software, Inc., San Jose, CA, USA) software packages. All tests were two-tailed and a *p*-value < 0.05 was considered to indicate statistical significance.

## 3. Results

### 3.1. TA-SCs and SCs Regulate the Migration, Invasion, and Proliferation of SCLC Cells In Vitro

**Figure 1:**
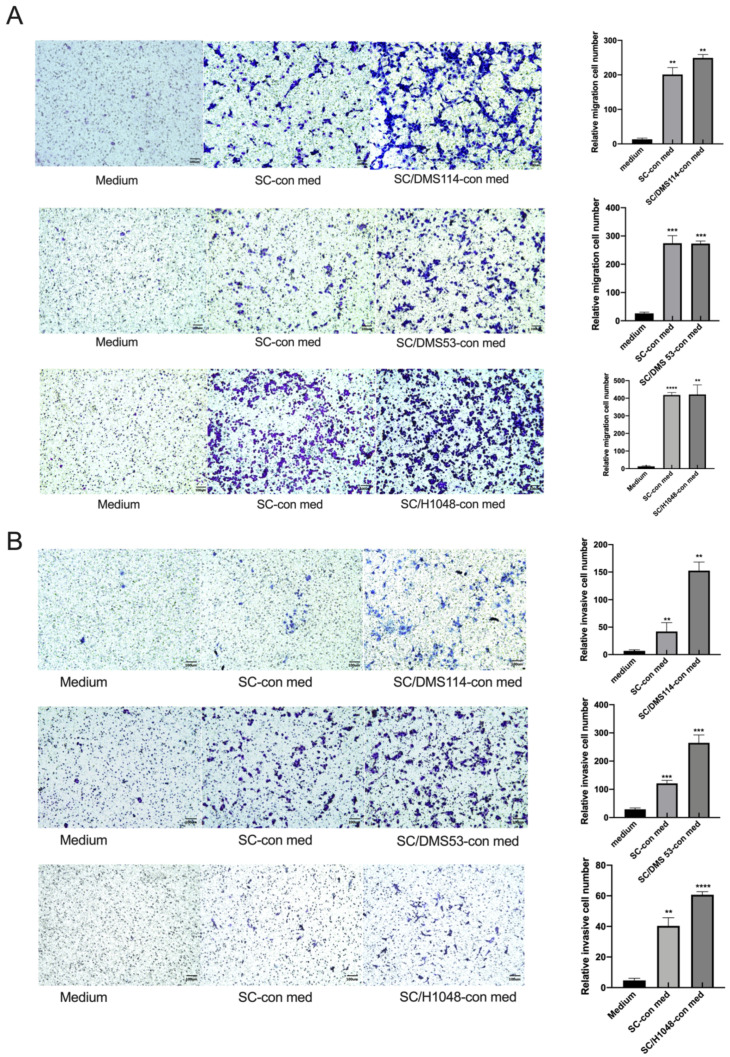
SCs increase the migration (**A**) and invasion (**B**) of small-cell lung cancer cells. DMS114, DMS53, and H1048 cells were cultured in Transwell inserts (8-μm pore size) without (migration assay) or with a Matrigel layer (invasion assay). The bottom chambers contained medium (medium) as a control, SC-conditioned medium (SC-con med), or conditioned medium from SCs pretreated with DMS114, DMS53, or H1048 cells (SC/DMS114-con, SC/DMS53-con medium, or SC/H1048-con medium). The tumor cells were allowed to transmigrate for 20 h in the migration assay and 40 h in the invasion assay at 37 °C. Cells that penetrated the underside surfaces of the membranes were fixed, stained, and counted on a grid in 10 HPF. Stained membranes from the representative experiment are shown. The results are shown as a relative invasion cell number—transmigrating to control cell ratio. Each assay was carried out in duplicates and all experiments were repeated at least three times. Scale bar is 100 um. **, *p* < 0.01, ***, *p* < 0.001, ****, *p* < 0.0001. SC/DMS114-con, SC/DMS53-con, SC/H1048-con medium, or SC-con vs. medium (one-way ANOVA).

**Figure 2:**
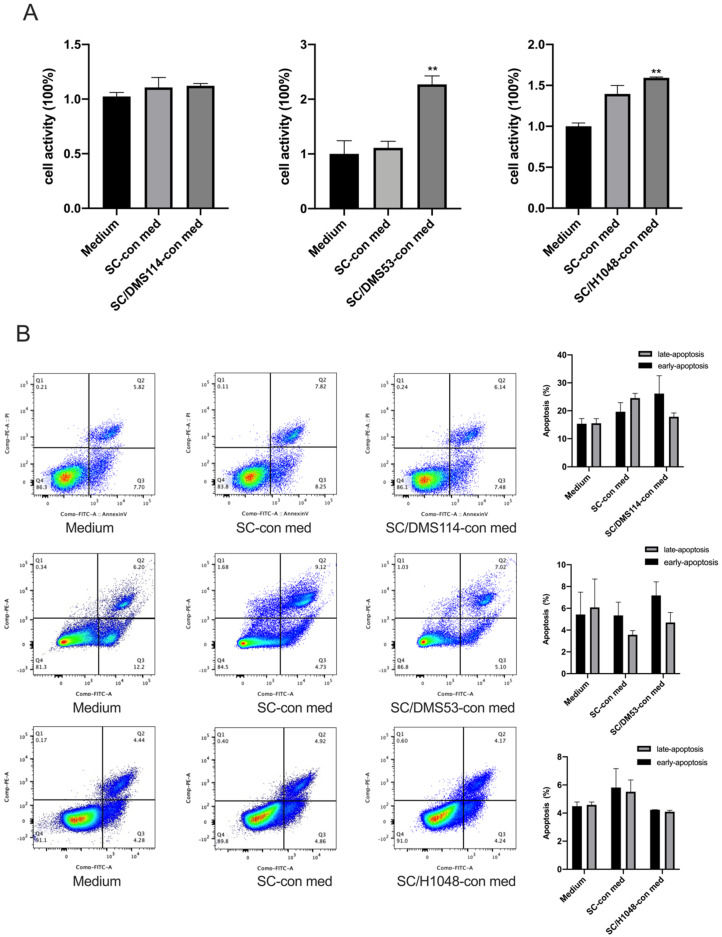
The SC effects on the SCLC cell proliferation and viability. (**A**) SCs may increase the proliferation of the SCLC cells in vitro. DMS114, DMS53, and H1048 cells were cultured for 24 h in a complete RPMI-1640 medium containing 10% (*v/v*) SC medium (medium), 10% (*v/v*) SC-conditioned medium (SC−con med), and 10% (*v/v*) conditioned medium from SCs pretreated with DMS114, DMS53, and H1048 cells (SC/DMS114-con med, SC/DMS53-con med, or SC/H1048-con med). Cell proliferation was determined with the cell counting-8 assay. The results are expressed as the mean ± SEM from three independent experiments. **, *p* < 0.01; SC/DMS53-con, or SC/H1048-con vs. medium. (**B**) SCs do not alter the apoptosis of the SCLC cells in vitro. DMS114, DMS53, and H1048 cells were cultured for 48 h in a complete RPMI medium containing 10% (*v/v*) SC medium (medium), 10% (*v/v*) SC-conditioned medium (SC-con med), and 10% (*v/v*) conditioned medium from SCs pretreated with DMS114, DMS53, or H1048 cells (SC/DMS114-con med, SC/DMS53-con med, or SC/H1048-con med). Then, cells were stained with PI and FITC-conjugated Annexin V, and analyzed by flow cytometry. Q1: necrotic, AV−/PI+; Q2: late-apoptotic, AV+/PI+; Q3: live, AV−/PI−; Q4: early-apoptotic, AV+/PI−. The results are expressed as the mean ± SEM from three independent experiments.

SCs were reported to facilitate the progression of different cancers, including NSCLC [13,17,30,31]. Analysis of the effect of SCs on the SCLC revealed that tumor-activated SCs and control SCs could significantly chemoattract different SCLC cell lines, including DMS114, DMS53, and H1048 cells. For example, the SC-conditioned medium attracted tumor cells at a rate of 200-fold greater than the control medium (*p* < 0.05, *n* = 4; Figure 1A). Next, the results of the Matrigel transwell invasion assay revealed that tumor-preactivated SCs activated the SCLC cells even stronger than the conditioned medium from the SC and control medium (*p* < 0.05, *n* = 4; Figure 1B). Furthermore, SCs demonstrated the ability to increase the proliferation of DMS53 and H1048, but not DMS114 cells in vitro (Figure 2A). However, neither control nor tumor-activated SCs up-regulated apoptosis of the SCLC cells in vitro (Figure 2B). Altogether, these results demonstrate that SCs can modify motility and the proliferative ability of the SCLC cells in vitro, without altering their viability.

### 3.2. TA-SCs and SCs Promote the Growth of SCLC Cells In Vivo

**Figure 3:**
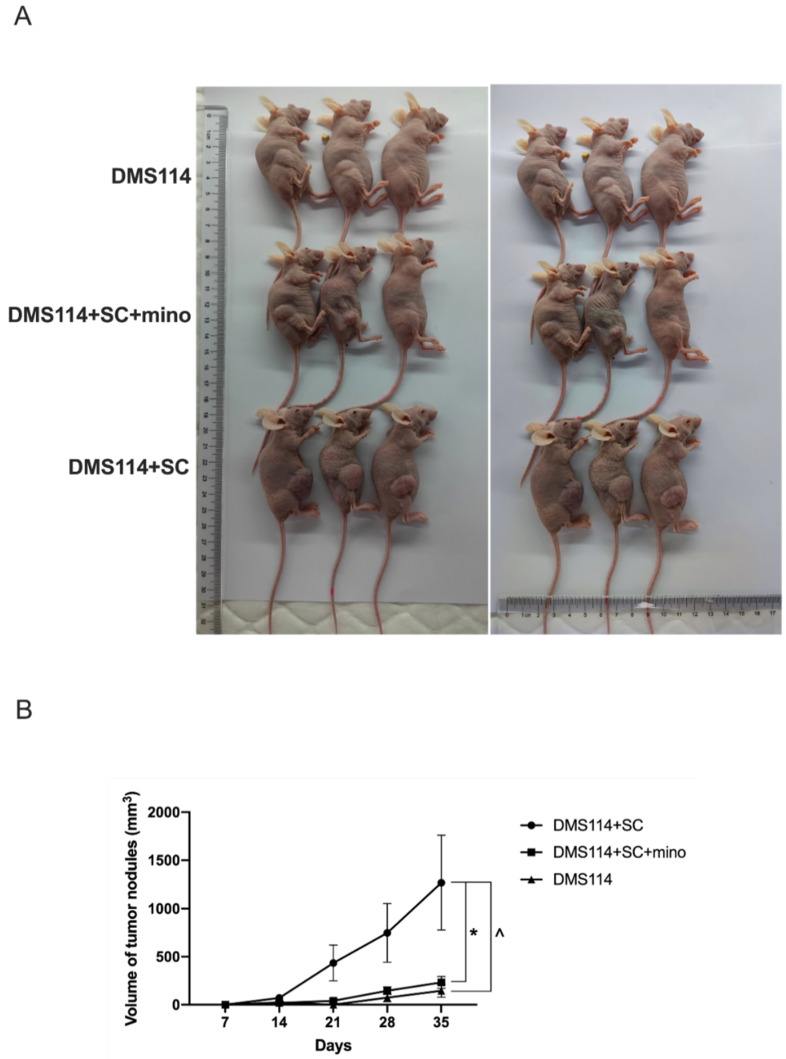
SCs promote tumorigenicity of DMS114 cells in vivo. Nude mice (*n* = 6/group) were injected with DMS114 cells (DMS114), DMS114 cells plus SCs (DMS114+SC), and DMS114 cells plus minocycline-pretreated SCs (DMS114+SC+mino). Tumor growth was assessed for five weeks. Representative pictures of three nude mice in each group are shown (**A**). Tumor growth curves of all six mice in each group are shown (**B**). The results are expressed as the mean ± SEM. *, *p* < 0.05, DMS114+SC vs. DMS114+SC+mino group; ^, and *p* < 0.05, DMS114+SC vs. DMS114 group.

After comparing and contrasting the effects of the control and the tumor-activated SC on three SCLC cell lines, we revealed that the tumor-activated SC demonstrated the strongest effect over the SC group and the control group on the migratory activity and invasiveness of DMS114 cells. To assess the effects of SCs on the growth of SCLC cells in vivo, mouse xenograft tumor models were established using DMS114 tumor cells combined with the control and minocycline-treated SCs in nude mice. Minocycline was chosen because it inhibits the tumor-dependent transdifferentiation and activation of signaling pathways in mature SCs in vitro and in vivo [13]. Our data shown in Figure 3A,B revealed that all mice had developed subcutaneous tumors 10 days after SCLC cell injection. Consistent with our in vitro results, we observed that the addition of SCs to injected SCLC cells dramatically accelerated tumor growth in vivo (*p* < 0.05). However, pre-treatment of SCs with minocycline abrogated the ability of SCs to accelerate tumor growth in mice (*p* < 0.05). These results suggest that in the tumor environment, SCs can be polarized into a protumorigenic repair-like phenotype and can contribute to SCLC growth in vivo. Thus, SCs seen in the tumor milieu may be defined as tumor-associated repair-like SCs (TAR-SCs).

### 3.3. Analysis of DEGs in the Cancer Cells from the TAR-SC Group, the SC Group, and the Control Group

**Figure 4:**
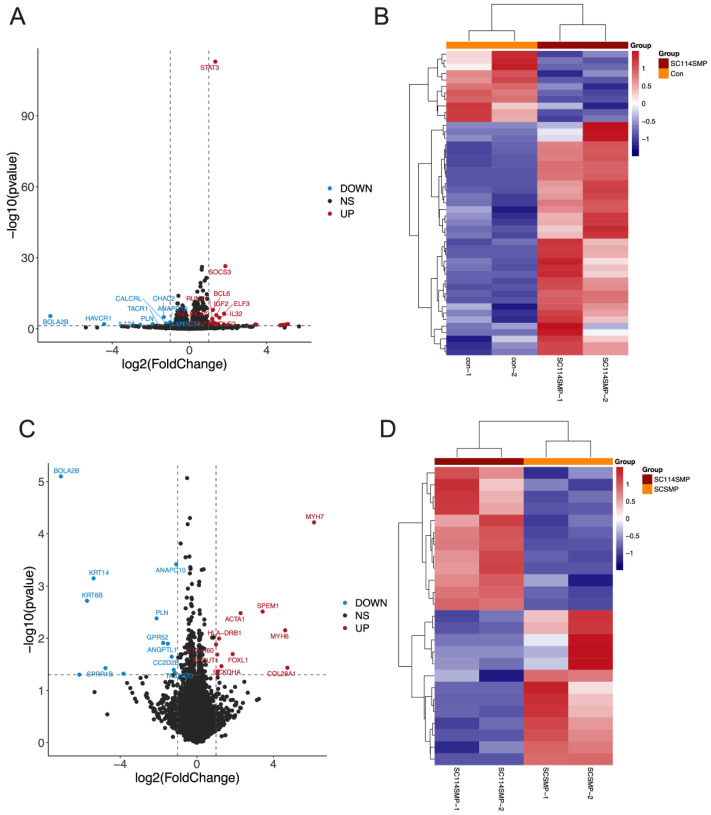

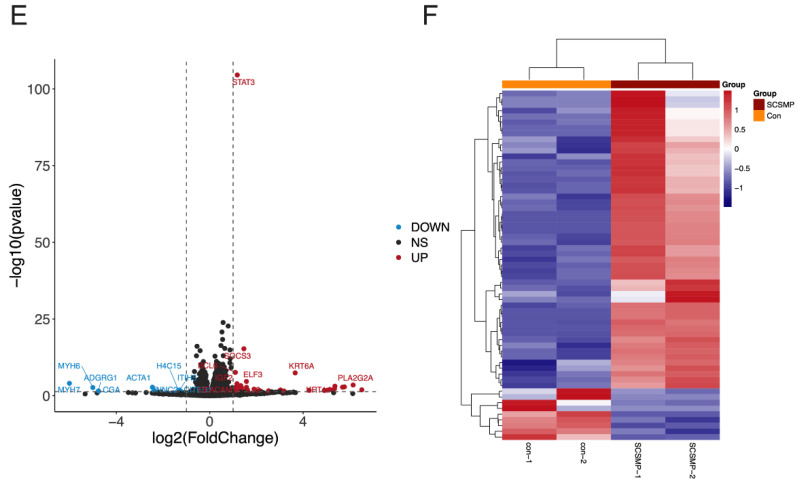
Expression profiles of distinct mRNAs in SC-treated SCLC cells. Volcano plot (**A**) and heat map (**B**) of the differentially expressed mRNAs in SCLC cells treated with the tumor-activated SC-conditioned medium (SC114MP-1 and SC114MP-2) and control medium (con-1 and con-2). Volcano plot (**C**) and heat map (**D**) of the differentially expressed mRNAs in SCLC cells treated with the tumor-activated SC-conditioned medium (SC114MP-1 and SC114MP-2) and SC-conditioned medium (SCSMP-1 and SCSMP-2). Volcano plot (**E**) and heat map (**F**) of the differentially expressed mRNAs in SCLC cells treated with the SC-conditioned medium (SCSMP-1 and SCSMP-2) and control medium (con-1 and con-2).

**Figure 5:**
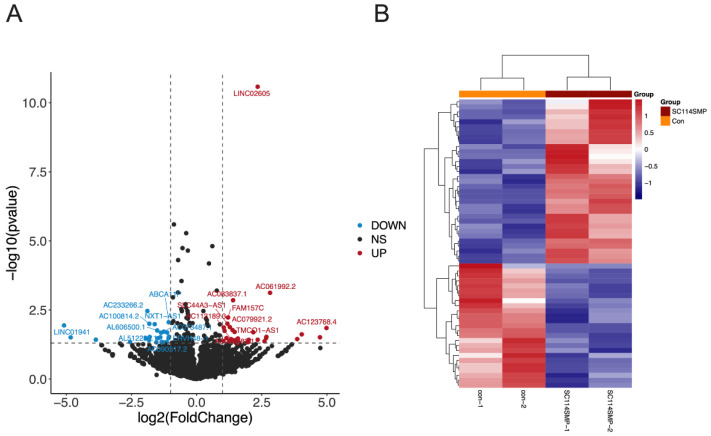

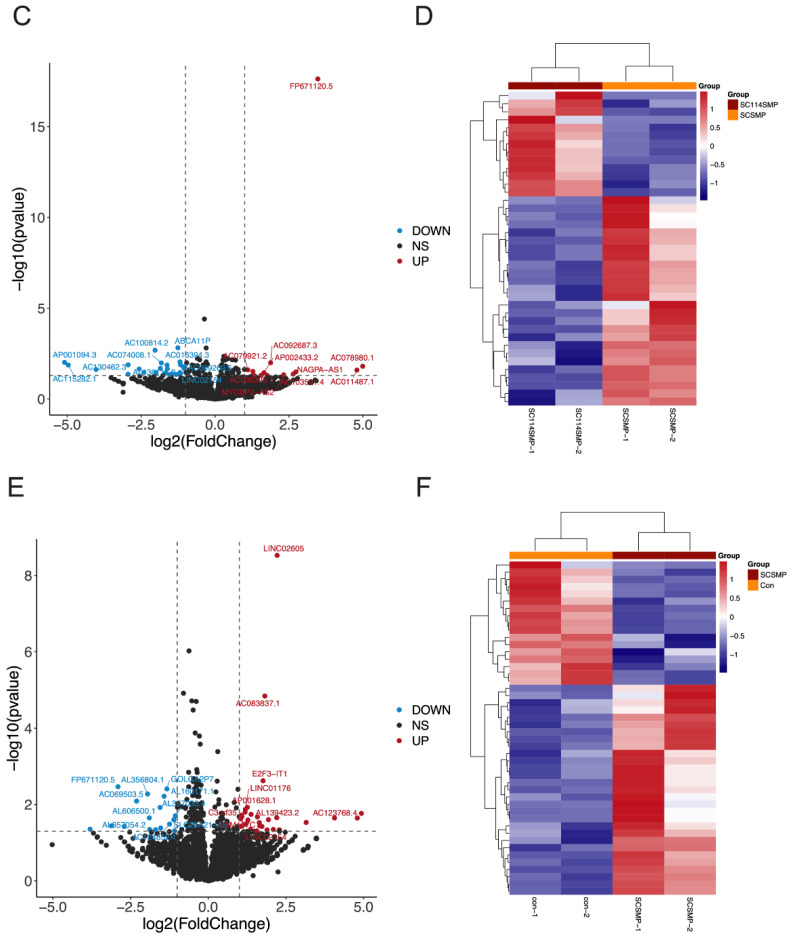
Expression profiles of distinct lncRNAs in SC-treated SCLC cells. Volcano plot (**A**) and heat map (**B**) of the differentially expressed lncRNAs in SCLC cells treated with the tumor-activated SC-conditioned medium (SC114MP-1 and SC114MP-2) and control medium (con-1 and con-2). Volcano plot (**C**) and heat map (**D**) of the differentially expressed lncRNAs in SCLC cells treated with the tumor-activated SC-conditioned medium (SC114MP-1 and SC114MP-2) and SC-conditioned medium (SCSMP-1 and SCSMP-2). Volcano plot (**E**) and heat map (**F**) of the differentially expressed lncRNAs in SCLC cells treated with the SC-conditioned medium (SCSMP-1 and SCSMP-2) and control medium (con-1 and con-2).

Next, we determined how SCs can alter gene expression in SCLC cells. DMS114 cells were treated with a tumor-activated SC-conditioned medium (TAR-SCs group), SC-conditioned medium (SC group), and medium (control group), and each group consisted of two samples: SC114SMP-1 and SC114SMP-2 (TAR-SC group), SCSMP-1 and SCSMP-2 (SC group), and con-1 and con-2 (control group). The sequencing of samples from the TAR-SC group, SC group, and control group generated the raw reads 81877352/87829546, 82415818/88307074, and 86519846/88152114, respectively. Overall, 17692 mRNA and 11583 lncRNA were identified. A DEseq analysis was performed to identify differentially expressed genes among the predicted novel mRNA and lncRNA. Comparing the TAR-SC and control groups, 15325 DEGs as mRNA (Figure 4A,B) and 7173 as lncRNA were identified (Figure 5A,B), with 47 significant DEGs as mRNA and 58 as lncRNA (Supplementary Table S2). Comparing the TAR-SC and SC groups, there were 15363 DEGs as mRNA (Figure 4C,D) and 7300 as lncRNA (Figure 5C,D), with 26 significant DEGs as mRNA and 40 as lncRNA (Supplementary Table S1). Comparing the SC and control groups, there were 15367 DEGs as mRNA (Figure 4E,F) and 7173 as lncRNA (Figure 5E,F), with 61 significant DEGs as mRNA and 46 as lncRNA (Supplementary Table S3).

### 3.4. GO Terms and the KEGG Pathway Analysis of DEGs

**Figure 6:**
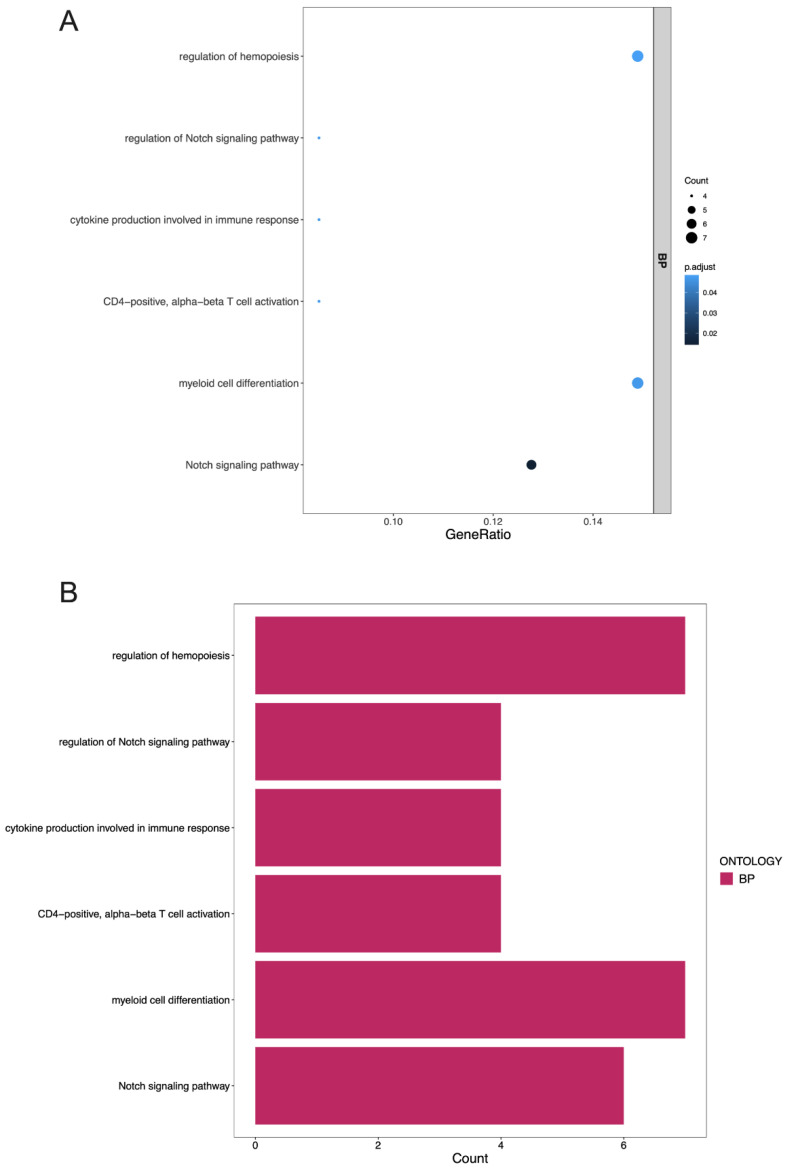

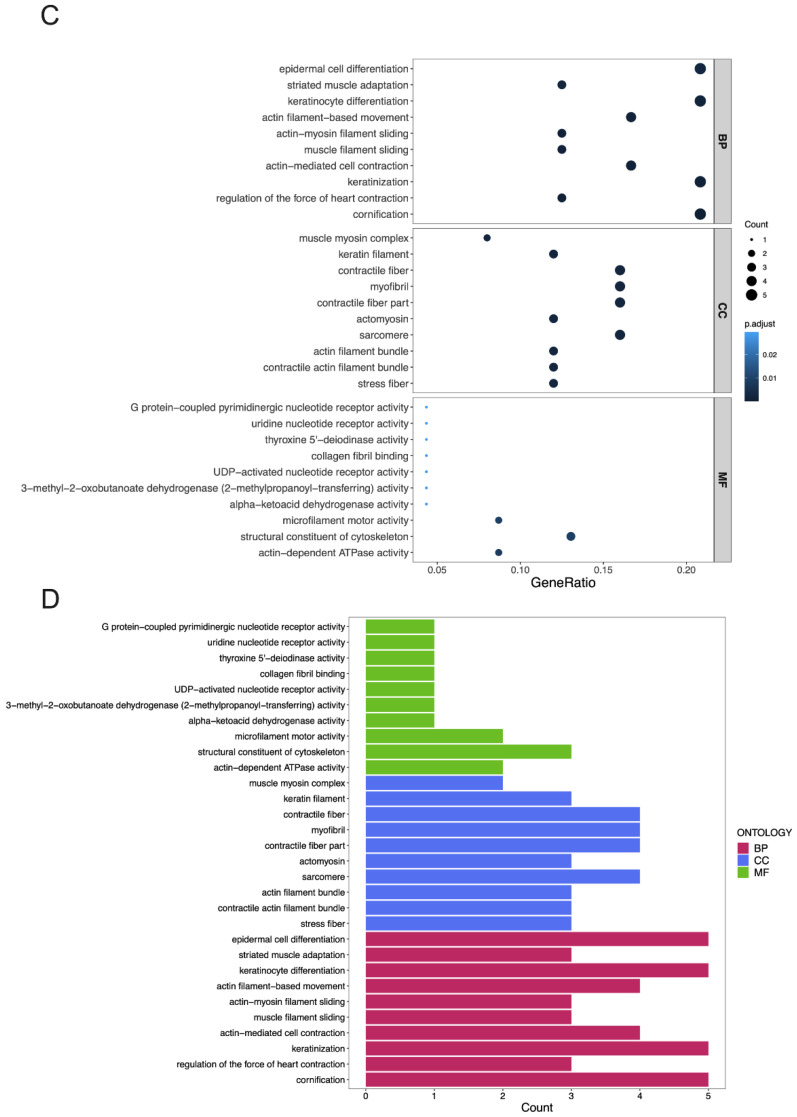

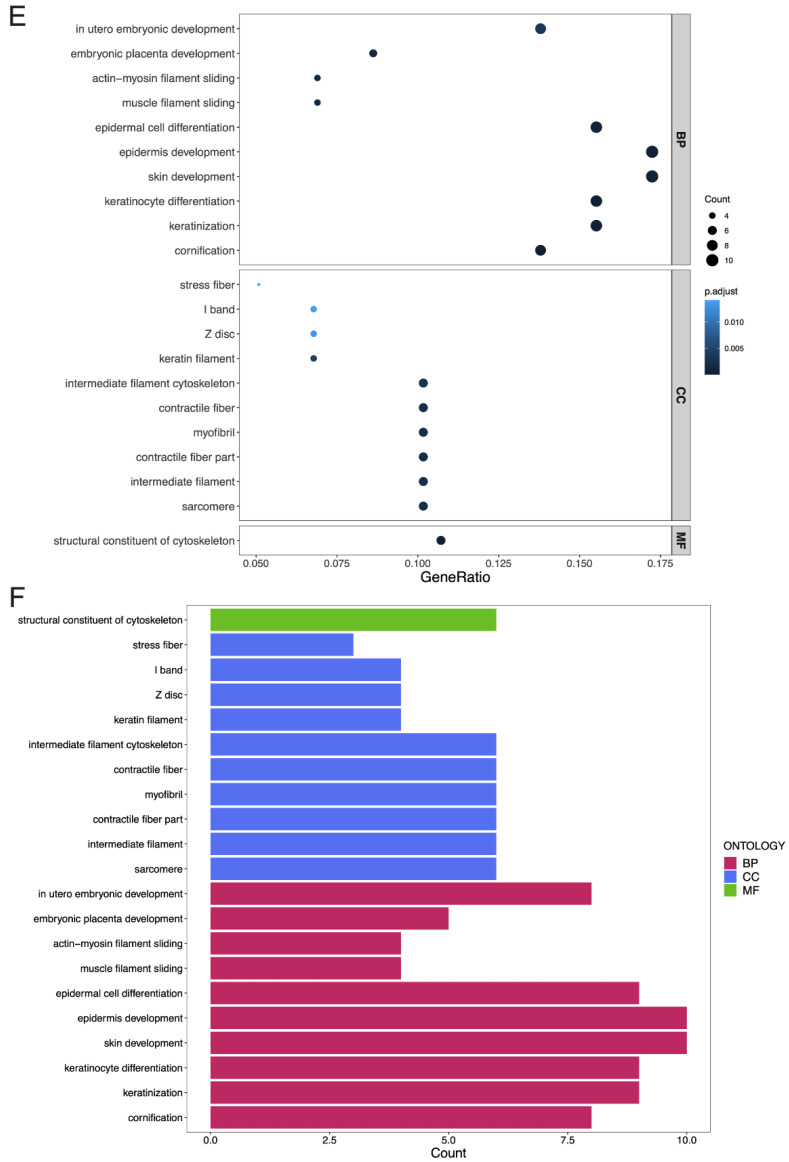
Gene ontology (GO) enrichment annotations of pathological progression of differentially expressed mRNAs in the SC-treated SCLC cells was categorized into three groups: biological process (BP), cellular component (CC) and molecular function (MF). (**A**,**B**): Enrichment result of GO of the differentially expressed mRNAs in treated SCLC cells comparing the SC/DMS114-conditioned group (SC114MP-1 and SC114MP-2) and control group (con-1 and con-2). (**C**,**D**): Enrichment result of GO of the differentially expressed mRNAs in treated SCLC cells comparing the SC/DMS114-conditioned group (SC114MP-1 and SC114MP-2) and SC-conditioned group (SCSMP-1 and SCSMP-2). (**E**,**F**): Enrichment result of GO of the differentially expressed mRNAs in treated SCLC cells comparing the SC-conditioned group (SCSMP-1 and SCSMP-2) and control group (con-1 and con-2).

The functional categories of the DEGs in the SC-treated SCLC cells were determined by GO terms and the KEGG pathway analysis. The top highly enriched GO terms of the biological process (BP), cellular component (CC), and molecular function (MF) categories are shown in Figure 6. The top terms of the DEGs in the TAR-SC group compared to the control group were most enriched in the BP category, especially the Notch signaling pathway (GO:0007219), myeloid cell differentiation (GO:0030099), and CD4-positive alpha-beta T-cell activation (GO:0035710). The top terms of the enriched genes in the TAR-SC group compared to the SC group were cornification (GO:0070268), regulation of the force of heart contraction (GO:0002026), and keratinization (GO:0031424) in the BP category; stress fiber (GO:0001725), contractile actin filament bundle (GO:0097517), and contractile fiber (GO:0043292) in the CC category; and actin-dependent ATPase activity (GO:0030898), structural constituent of the cytoskeleton (GO:0005200), and microfilament motor activity (GO:0000146) in the MF category. The most significant terms in the SC group compared to the control group were enriched in cornification (GO:0070268), keratinization (GO:0031424), and keratinocyte differentiation (GO:0030216) in the BP category; sarcomere (GO:0030017), intermediate filament (GO:0005882), and contractile fiber part (GO:0044449) in the CC category; and the structural constituent of the cytoskeleton (GO:0005200) in the MF category.

**Figure 7:**
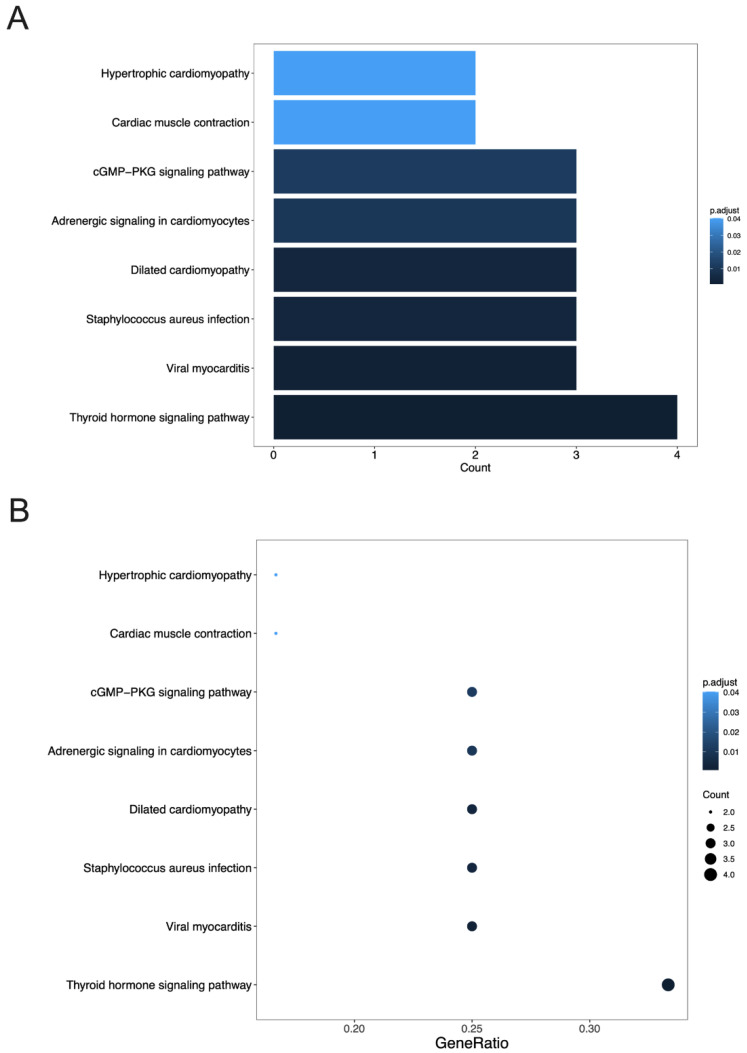

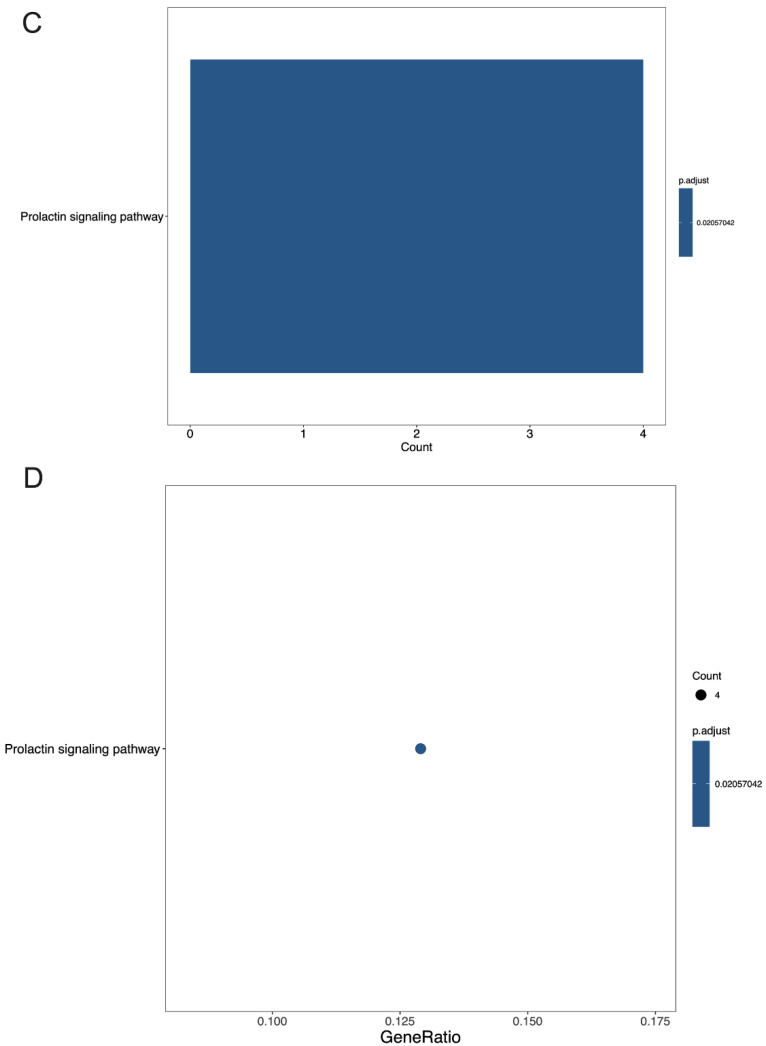
The Kyoto Encyclopedia of Genes and Genomes (KEGG) pathway enrichment analysis of the differentially expressed mRNAs in treated SCLC cells compared to the SC/DMS114-conditioned group, SC-conditioned group, and control group. (**A**,**B**): Enrichment result of the KEGG analysis of the differentially expressed mRNAs in SCLC cells treated by the tumor-activated SC-conditioned medium (SC114MP-1 and SC114MP-2) and SC-conditioned medium (SCSMP-1 and SCSMP-2). (**C**,**D**): Enrichment result of the KEGG analysis of the differentially expressed mRNAs in SCLC cells treated by the SC-conditioned group (SCSMP-1 and SCSMP-2) and control medium (con-1 and con-2).

The KEGG pathway enrichment analysis was conducted to identify the signaling pathways of the significant DEGs using an adjusted *p*-value of <0.05 as the threshold value. The results showed that none of the DEGs were significantly enriched when compared to the TAR-SC and control groups. Comparing the TAR-SC and SC groups, the following top six significantly enriched pathways were identified (Figure 7): the thyroid hormone signaling pathway, viral myocarditis, staphylococcus aureus infection, dilated cardiomyopathy, adrenergic signaling in cardiomyocytes, and the cGMP-PKG signaling pathway. The significant terms comparing the SC and control groups were enriched in the prolactin signaling pathway.

### 3.5. Construction of Circular RNA-Associated ceRNA Networks

**Figure 8:**
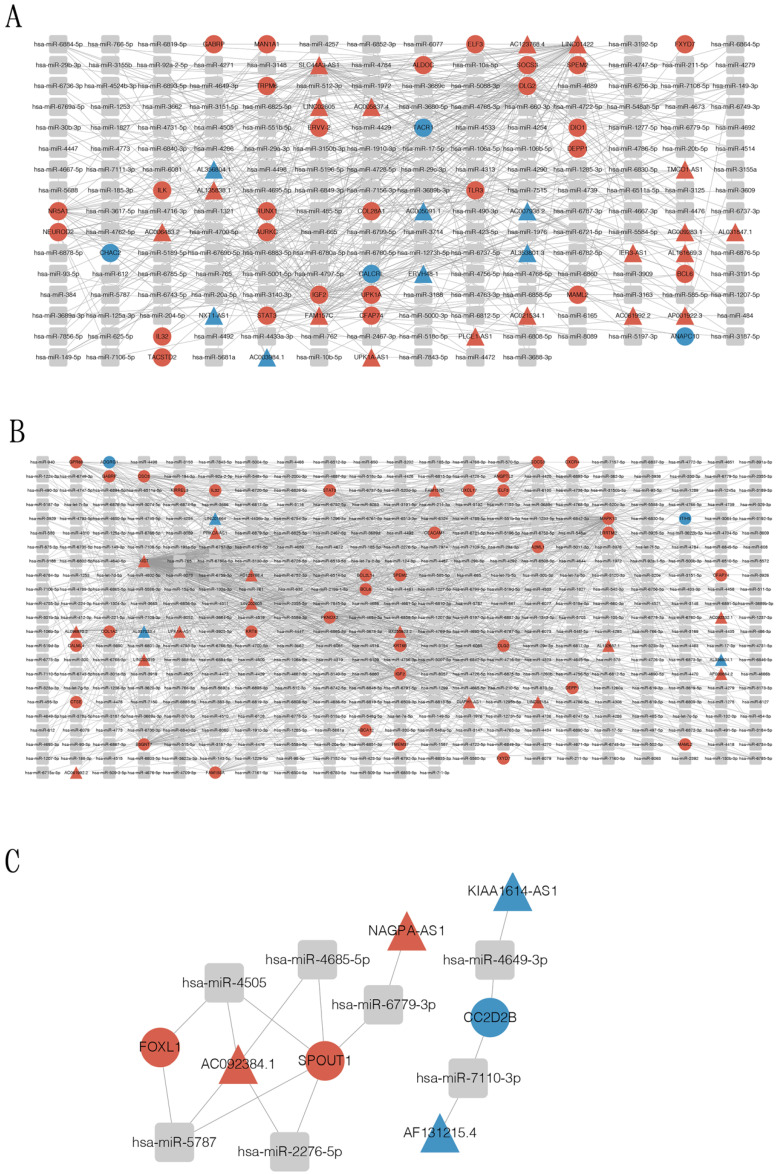
mRNA-miRNA-lncRNA and ceRNA networks in small-cell lung cancer, conditioned by SCs. According to the targeting relationship between miRNA-mRNA and miRNA-lncRNA, and the expression trend of lncRNA and mRNA, mRNA-miRNA-lncRNA and ceRNA networks between the SC/DMS114-conditioned group and control group (**A**), the SC-conditioned group and control group (**B**), and the SC/DMS114-conditioned group and SC-conditioned group (**C**) were conducted. The gray rectangle represents miRNA, the circle represents mRNA, the triangle represents lncRNA, the red color represents the gene up-regulation in the corresponding group comparison, and the blue color represents the down-regulation.

To elucidate the competitive endogenous RNA (ceRNA)-associated mechanisms of SC-induced SCLC activation, mRNA-miRNA-lncRNA, and ceRNA networks were constructed to compare the effect of the tumor-activated and control SCs on SCLC cells (Figure 8). Analysis was conducted according to the targeting relationship between miRNA-mRNA, miRNA-lncRNA, and the association of different lncRNA with mRNA. A predictive analysis was performed using Miranda software (Tot_Energy ≤ −20, Total_Score ≥ 180). We also explored reported miRNA based on target mRNA collecting miRNA-mRNA pairs using available databeses—Targetscan, miRDB, mirTarBase, and miRwalk. Next, selected miRNAs were used to identify the target lncRNAs utilizing the lncBaseV2 database and Miranda software. A total of 2372 miRNA-mRNA pairs and 687 miRNA-lncRNA pairs, 343 miRNA-mRNA pairs and 129 miRNA-lncRNA pairs, and 3835 miRNA-mRNA pairs and 1089 miRNA-lncRNA pairs were included in the ceRNA networks of the TAR-SC versus control group analysis, the SC versus control group analysis, and the TAR-SC versus SC group analysis, respectively. In addition, we identified 394 pairing links of mRNA-miRNA-lncRNA in the TAR-SC and control groups, 10 pairs in the SC and control groups, and 955 pairs in the TAR-SC and SC groups.

In the TAR-SC and control groups, the core lncRNAs include FAM157C, SLC44A3-AS1, and LINC00896, which could competitively target transcription of STAT3, SOCS3, EUROD2, IGF2, DLG2, BCL6, and RUNX1. In the SC and control groups, the core lncRNAs include XIST, FAM157C, and LINC02605, which could competitively target transcription of STAT3, SOCS3, GABRP, IGF2, ELF3, CEACAM1, and BCL6. In the TAR-SC and SC groups, the core lncRNA includes AC092384.1, which could competitively target the transcription of FOXL1 and SPOUT1, which were not significant DEGs. Thus, by comprehensively integrating gene and miRNA expression data of the neuroglial SCLC network (Figure 8), our data have established the SCLC-SC crosstalk-related mRNA-miRNA-lncRNA competitive network for the first time. Further optimization of the mRNA-lncRNA relationship analysis in the SCLC and systematic dissection of lncRNA-associated ceRNA networks would lead to the identification of neurological risk markers reflecting the dysregulation of SCLC-associated pathobiological processes.

## 4. Discussion

Emerging evidence shows that Schwann cells may coordinate neural and neuroimmune communications with the malignant cells in the tumor microenvironment, controlling tumor growth, spreading, and metastasis [32,33,34]. Tumor-induced activation, dedifferentiation, and denervation of SCs are well-established phenotypic characteristics of so-called “repair” neurotrauma-associated SCs. At the same time, this characterizes tumor-associated SCs [13], and such alterations of SCs correlate with tumor progression and metastasis. Our results here demonstrate that SCs, specifically tumor-activated (or tumor-associated) TA-SCs, promoted the migration and invasion properties of SCLC cells in several in vitro assays and supported tumor growth if co-administered with malignant cells in mice in vivo. Early studies revealed that melanoma-activated SCs, functionally and phenotypically resembling the neurotrauma-associated repair SCs, could promote tumor growth in vitro and in vivo by releasing different cytokines and chemokines and modulating the tumor immunoenvironment [13,14,35]. Tumor-induced production of CXCL5 by SCs supported lung cancer spreading, epithelial-mesenchymal transition (EMT), and formation of metastasis in vivo [17], while SC-derived CCL2 promoted the M2 polarization of macrophages and lung cancer cell proliferation [18]. Furthermore, SC-produced CCL2 was shown to promote the proliferation, migration, invasion, and EMT of cervical cancer cells [36]. However, much less is known about molecular mechanisms regulating malignant cell activity and function by tumor-activated or “repair-like” SCs.

Our results revealed that the treatment of different SCLC cell lines with SC-derived factors significantly altered the expression of several genes in tumor cells. For instance, tumor-activated SCs up-regulated the expression of the STAT3, SOCS3, BCL6, ELF3, IGF2, IL32, RUNX1, and NEUROD2 genes and inhibited the expression of the CHAC2 and BOLA2B genes in SCLC cells in vitro. The STAT3 transcription factor is involved in many biological processes, including cell proliferation, survival, differentiation, and angiogenesis [37,38]. Aberrantly activated STAT3 can induce immunosuppression by regulating crosstalk between tumor cells and immune cells in the tumor microenvironment. In tumor cells, hyperactivated STAT3 promotes the expression of immunosuppressive factors such as VEGF, IL-6, and IL-10 [39]. At the same time, these tumor-derived factors may serve as STAT3 activators in the tumor milieu, enhancing STAT3 signaling in various immune cell subsets and tumor-associated fibroblasts [40]. The SOCS3 gene encodes a protein from the STAT-induced STAT inhibitor family, which consists of several cytokine-inducible negative regulators of cytokine signaling. Epigenetic alterations and post-transcriptional modifications of the SOCS family genes, such as promoter methylation and miRNA regulation, are the main causes of gene silencing in cancer. SOCS3 hypermethylation has been observed in cholangiocarcinoma and head and neck squamous cell carcinoma [41,42]. The transcription factor RUNX1 and cofactor CBFB form a complex that promotes EMT in renal carcinoma [43] and is elevated upon EMT in endometrial cancer [44]. RUNX1 maintains the active/phosphorylated form of the oncogene STAT3 by inhibiting SOCS3/4 in skin cancer, thereby increasing cell survival, proliferation, and invasion [45]. Alteration of these gene expressions in cancerous cells by SCs has never been reported.

We demonstrated that the expression of both STAT3 and SOCS3 was up-regulated in DMS114 tumor cells pre-incubated with the tumor-activated SC-conditioned medium. This allows speculation that up-regulation of STAT3 and activation of the NF-κB pathway in malignant cells may result in the significant up-regulation of tumor invasiveness and, thus, tumor progression. SOCS3 can terminate the JAK/STAT3 pathway activation downstream of the STAT3 pathway, and up-regulation of the STAT3 pathway could lead to the overexpression of SOCS3. Activation of the JAK/STAT signaling pathway has been linked to pancreatitis and perineural invasion associated with poor prognosis in pancreatic cancer [46]. Furthermore, our data indicate that in the tumor microenvironment SCs can be polarized into TAR-SCs responsible for the up-regulation of SCLC proliferation, growth, and invasion. Together, our results revealed, for the first time, potential molecular mechanisms of the protumorigenic activity of SCs in the tumor microenvironment.

Notably, both control and tumor-treated SCs were able to alter gene expression in SCLC cells, although the effect of tumor-activated SCs on the expression of several genes, including STAT3, was significantly stronger than the effect of intact SCs. For instance, incubation of SCLC cells in the activated SC-conditioned medium up-regulated expression of the STAT3, SOCS3, BCL6, ELF3, IGF2, and IL32 genes, in addition to the KRT6A, PLA2G2A, CEACAM1, KRT14, SPEM2, GABRP, and A2M genes, while the expression of the ACTA1 and MYH7 genes was down-regulated. Interestingly, the effect of the tumor-activated versus control SCs on SCLC cell invasiveness was significantly higher, and this was associated with a higher level of expression of several genes, including the STAT3, SOCS3, BCL6, ELF3, IGF2, and IL32 genes, in SCLC cells. Together, this supports our suggestion about the involvement of these genes in the promotion of SCLC invasiveness and motility by TAR-SCs in the tumor microenvironment. Further analysis of the functional expression of different signaling proteins in the malignant cells and their correlation with tumor cell behavior in vitro and in vivo is needed to validate the involvement of SCs in carcinogenesis and determine whether tumor-associated SCs could be targeted for therapeutic purposes to modify the tumor microenvironment.

It is also important to note that combining gene expression data with gene ontology (GO) annotations to rank and visualize genes demonstrated clustering in the Notch signaling pathway (GO:0007219) and regulation of the Notch signaling pathway (GO:0008593) in SC-treated SCLC cells. The Notch signaling pathway regulates a variety of cellular processes, including proliferation, stem cell maintenance, differentiation, and cell death [47]. The carcinogenic effects of Notch signaling include the inhibition of cell apoptosis, the promotion of drug resistance, the induction of EMT and angiogenesis, the maintenance of the stem-like phenotype, and the promotion of metastasis [48,49]. It was reported that inactivating mutations in the Notch family genes have been identified in 25% of human SCLC cases [50]. In addition, the Notch signaling pathway, activated in injured nerves, was reported to drive myelinating SCs toward demyelination [51]. Our results indicate that the Notch signaling pathway was significantly up-regulated in DMS114 cells cultured with the SC-conditioned medium and particularly with the tumor-activated SC-conditioned medium. As this was associated with a significant up-regulation of SCLC cell migration and invasion, it is intriguing to speculate that this pathway of tumor neuroregulation should be additionally verified as a novel therapeutic target in vivo.

The complexity and behavior of the competing endogenous RNA (ceRNA) networks has never been characterized in the SC-treated cancerous cells. Our novel results suggest a potential involvement of FOXL1, SPOUT, and NAGPA-AS1, and related lncRNA AC092384.1 in SCLC cell regulation by SCs. The FOXL1 transcription factor regulates epithelial proliferation and could promote different types of cancer, including lung cancer [52,53]. However, the role of FOXL1 in the PNS-supported SCLC development and progression remains to be elucidated. Our results also suggest that lncRNA AC092384.1 may be a key lncRNA in SCLC cells regulated by TAR-SCs. Further detailed research is required to validate this hypothesis.

In summary, our findings demonstrate that SCs, particularly TAR-SCs, may substantially promote the progression of SCLC and reveal the primary molecular mechanisms of this new phenomenon. Further analyses of SC-derived factors, mechanisms of SC activation, and validation of the clinical utility of harnessing SC activity in the tumor milieu are required. For instance, one approach to inhibiting SCs in the SCLC environment may rely on the treatment of SC-derived tumors—neurofibromatosis. This process involves the use of selumetinib, a potent, highly selective MEK inhibitor [54]. Animal experiments verifying its efficacy in lung cancer models are in progress in our laboratory.

## 5. Conclusions

In conclusion, we revealed that tumor-associated Schwann cells may facilitate the invasion and progression of SCLC. Analysis of potential underlying mechanisms allowed the construction of mRNA-miRNA-lncRNA networks in tumor cells stimulated by differentially activated SCs. These newly identified networks provide valuable insights into the role of SCs in tumor progression and reveal potential biomarkers and/or therapeutic targets of the cancer-nerve/SC axis that may have a marked impact on SCLC therapy.

## Data Availability

The data presented in this study are available on request from the corresponding author.

## Supplementary Materials

The following supporting information can be downloaded at: https://www.mdpi.com/article/10.3390/cancers14246132/s1, Figure S1: full-sized Figure 8: mRNA-miRNA-lncRNA ceRNA networks between SC/DMS114-conditioned group and control group (**A**), SC-conditioned group and control group (**B**), Table S1: IDs of differentially expressed mRNAs and lncRNA of TAR-SC vs. SC groups, Table S2: IDs of differentially expressed mRNAs and lncRNA of TAR-SC vs. control groups. Table S3: IDs of differentially expressed mRNAs and lncRNA of SC vs. control groups.
