# Supplementary material for: A Novel Therapeutic Target for Small-Cell Lung Cancer: Tumor-Associated Repair-like Schwann Cells
Source: Cancers (Basel). 2022 Dec 12;14(24):6132. doi: 10.3390/cancers14246132 (PMC9776631; doi:10.3390/cancers14246132)
Supplement: Supplementary file 1 [file cancers-14-06132-s001.zip › cancers-1851146-supplementary.pdf]

cancers

MDPI

Supplementary Table S1. IDs of differentially expressed mRNAs and lncRNA of TAR-SC vs. SC groups.

DEGs of mRNA		DEGs of lncRNA	
Gene	Gene ID	Gene	Gene ID
DIO1	ENSG00000211452	AL136115.2	ENSG00000269967
SPRR3	ENSG00000163209	AC103591.4	ENSG00000285928
SPRR1B	ENSG00000169469	AL449283.1	ENSG00000229052
GPR52	ENSG00000203737	KIAA1614-AS1	ENSG00000232586
ANGPTL1	ENSG00000116194	AC092687.3	ENSG00000272275
ACTA1	ENSG00000143632	AC007098.1	ENSG00000231609
P2RY14	ENSG00000174944	AC074008.1	ENSG00000284902
ANAPC10	ENSG00000164162	AC010731.3	ENSG00000228577
HLA-DRB1	ENSG00000196126	AC012076.2	ENSG00000286864
PLN	ENSG00000198523	AC115282.1	ENSG00000286353
COL28A1	ENSG00000215018	AC078980.1	ENSG00000242029
SPOUT1	ENSG00000198917	ABCA11P	ENSG00000251595
QRFP	ENSG00000188710	AC079921.2	ENSG00000249685
CC2D2B	ENSG00000188649	AL512283.1	ENSG00000272472
TAS2R20	ENSG00000255837	LINC02154	ENSG00000235385
C12orf60	ENSG00000182993	AF131215.4	ENSG00000254936
KRT6B	ENSG00000185479	AC100814.2	ENSG00000272010
MYH6	ENSG00000197616	AL356481.3	ENSG00000280758
MYH7	ENSG00000092054	AP002433.2	ENSG00000285696
BOLA2B	ENSG00000169627	AL358216.1	ENSG00000225140
FOXL1	ENSG00000176678	AC016394.3	ENSG00000272599
SPEM1	ENSG00000181323	AC006206.2	ENSG00000256417
KRT13	ENSG00000171401	LINC02468	ENSG00000256499
KRT14	ENSG00000186847	MYCBP2-AS2	ENSG00000229521
BCKDHA	ENSG00000248098	AC066613.2	ENSG00000259678
		AC018926.3	ENSG00000277548
		AC012404.2	ENSG00000259575
		AC009269.5	ENSG00000274297
		NAGPA-AS1	ENSG00000267072
		AC130462.3	ENSG00000261864
		AC092384.1	ENSG00000205018
		AC104982.2	ENSG00000263477
		AC015802.3	ENSG00000267543

	AP001094.3	ENSG00000266149
	AC011445.2	ENSG00000269246
	AC005393.1	ENSG00000276445
	AC011487.1	ENSG00000213777
	FP671120.5	ENSG00000278996
	AP000962.3	ENSG00000287066

Supplementary Table S2. IDs of differentially expressed mRNAs and lncRNA of TAR-SC vs. control groups.

DEGs of mRNA		DEGs of lncRNA	
Gene	Gene ID	Gene	Gene ID
CFAP74	ENSG00000142609	AL031847.1	ENSG00000226944
DIO1	ENSG00000211452	ZPLD2P	ENSG00000236155
TACSTD2	ENSG00000184292	AL136115.2	ENSG00000269967
H3C14	ENSG00000203811	SLC44A3-AS1	ENSG00000224081
APOA2	ENSG00000158874	AL606500.1	ENSG00000287064
ELF3	ENSG00000163435	TMCO1-AS1	ENSG00000224358
CHAC2	ENSG00000143942	AC233266.2	ENSG00000261600
TACR1	ENSG00000115353	LINC01941	ENSG00000226813
LYG1	ENSG00000144214	ABCA11P	ENSG00000251595
CALCRL	ENSG00000064989	AC079921.2	ENSG00000249685
LXN	ENSG00000079257	AC096736.2	ENSG00000249479
IL12A	ENSG00000168811	PCDHGB8P	ENSG00000248449
BCL6	ENSG00000113916	E2F3-IT1	ENSG00000224707
ANAPC10	ENSG00000164162	AL021918.5	ENSG00000286652
TLR3	ENSG00000164342	IER3-AS1	ENSG00000272273
HAVCR1	ENSG00000113249	AL512283.1	ENSG00000272472
GABRP	ENSG00000094755	AC091729.1	ENSG00000224079
PLN	ENSG00000198523	AC006483.2	ENSG00000272719
MAN1A1	ENSG00000111885	AC005091.1	ENSG00000229893
COL28A1	ENSG00000215018	AC003984.1	ENSG00000235139
GDPD2	ENSG00000130055	AC007938.2	ENSG00000270823
TRPM6	ENSG00000119121	SDAD1P1	ENSG00000228451
NR5A1	ENSG00000136931	AC100814.2	ENSG00000272010
IGF2	ENSG00000167244	AC083837.1	ENSG00000285744
ILK	ENSG00000166333	LINC02605	ENSG00000261618
DLG2	ENSG00000150672	AP001922.3	ENSG00000254826
MAML2	ENSG00000184384	AL353801.3	ENSG00000273363
DEPP1	ENSG00000165507	PLCE1-AS1	ENSG00000268894
A2M	ENSG00000175899	AC048344.4	ENSG00000277342
DHRS2	ENSG00000100867	AC073487.1	ENSG00000257509
IL32	ENSG00000008517	AC078983.1	ENSG00000286563
NPIPB3	ENSG00000169246	AL356804.1	ENSG00000259033

BOLA2B	ENSG00000169627	AL135838.1	ENSG00000258620
SPEM1	ENSG00000181323	AL161669.3	ENSG00000278071
SPEM2	ENSG00000184560	AC123768.4	ENSG00000276724
ALDOC	ENSG00000109107	AC011330.2	ENSG00000275601
TBC1D3B	ENSG00000274808	AC021752.1	ENSG00000273674
NEUROD2	ENSG00000171532	AC090517.2	ENSG00000274667
KRT19	ENSG00000171345	AC012409.1	ENSG00000259359
STAT3	ENSG00000168610	FAM157C	ENSG00000260528
SOCS3	ENSG00000184557	AC113189.3	ENSG00000263301
FXYD7	ENSG00000221946	AC016876.4	ENSG00000276384
UPK1A	ENSG00000105668	AC009283.1	ENSG00000273576
SYT3	ENSG00000213023	AC005837.4	ENSG00000277382
ERVV-2	ENSG00000268964	AC061992.2	ENSG00000266970
AURKC	ENSG00000105146	AC021534.1	ENSG00000266711
RUNX1	ENSG00000159216	LINC02582	ENSG00000261780
		AC068473.2	ENSG00000266901
		NXT1-AS1	ENSG00000234832
		AL049539.1	ENSG00000275576
		AC011472.4	ENSG00000273733
		UPK1A-AS1	ENSG00000226510
		AC005393.1	ENSG00000276445
		LINC00896	ENSG00000236499
		LINC01422	ENSG00000223704
		FP236383.4	ENSG00000280614
		AL109761.1	ENSG00000244676
		ERVH48-1	ENSG00000233056

Supplementary Table S3. IDs of differentially expressed mRNAs and lncRNA of SC vs. control groups.

DEGs of mRNA		DEGs of lncRNA	
Gene	Gene ID	Gene	Gene ID
CFAP74	ENSG00000142609	AL139423.2	ENSG00000287727
PLA2G2A	ENSG00000188257	AL160171.1	ENSG00000285923
H4C15	ENSG00000270276	AL606500.1	ENSG00000287064
ELF3	ENSG00000163435	AC233266.2	ENSG00000261600
CTSE	ENSG00000196188	C3orf35	ENSG00000198590
ACTA1	ENSG00000143632	LINC02019	ENSG00000273356
TMEM37	ENSG00000171227	AC124852.1	ENSG00000248994

CXCR4	ENSG00000121966	AC008972.2	ENSG00000272081
ABCA12	ENSG00000144452	PCDHGB8P	ENSG00000248449
B3GNT7	ENSG00000156966	DIAPH1-AS1	ENSG00000246422
LRRIQ4	ENSG00000188306	AL357054.2	ENSG00000271727
BCL6	ENSG00000113916	AL138824.1	ENSG00000286885
LRRTM2	ENSG00000146006	E2F3-IT1	ENSG00000224707
GABRP	ENSG00000094755	AL355304.1	ENSG00000232618
FAM153A	ENSG00000170074	LINC01176	ENSG00000281404
GTF2H4	ENSG00000213780	LINC02154	ENSG00000235385
MAPK13	ENSG00000156711	XIST	ENSG00000229807
CGA	ENSG00000135346	AC083837.1	ENSG00000285744
COL1A2	ENSG00000164692	LINC02605	ENSG00000261618
SLC13A4	ENSG00000164707	BX255923.2	ENSG00000276462
CITED1	ENSG00000125931	AP000942.5	ENSG00000288528
ANGPTL2	ENSG00000136859	AL132657.1	ENSG00000226578
LCN2	ENSG00000148346	AL359844.1	ENSG00000285871
IGF2	ENSG00000167244	AC069503.5	ENSG00000287493
DLG2	ENSG00000150672	AL096870.2	ENSG00000260669
MAML2	ENSG00000184384	SLC25A21-AS1	ENSG00000258708
MMP3	ENSG00000149968	AL356804.1	ENSG00000259033
OR8B3	ENSG00000284609	AC123768.4	ENSG00000276724
PKNOX2	ENSG00000165495	GOLGA2P7	ENSG00000225151
KIRREL3	ENSG00000149571	NAGPA-AS1	ENSG00000267072
ITIH5	ENSG00000123243	AC074051.5	ENSG00000285567
DEPP1	ENSG00000165507	AC009141.1	ENSG00000286894
A2ML1	ENSG00000166535	AC092332.1	ENSG00000260064
A2M	ENSG00000175899	FAM157C	ENSG00000260528
BCL2L14	ENSG00000121380	ALOX12P2	ENSG00000262943
KRT6B	ENSG00000185479	PRKCA-AS1	ENSG00000264630
KRT6A	ENSG00000205420	AC061992.2	ENSG00000266970
KRT8	ENSG00000170421	AC087741.2	ENSG00000275479
MYH6	ENSG00000197616	AP000864.2	ENSG00000272788
MYH7	ENSG00000092054	AL357033.4	ENSG00000277496
GPR68	ENSG00000119714	UPK1A-AS1	ENSG00000226510
CALML4	ENSG00000129007	LINC01664	ENSG00000235478
IL32	ENSG00000008517	FP671120.5	ENSG00000278996

ADGRG1	ENSG00000205336	FP236383.4	ENSG00000280614
IRF8	ENSG00000140968	AP000962.3	ENSG00000287066
SPEM2	ENSG00000184560	AP001628.1	ENSG00000225218
FBXW10	ENSG00000171931		
TBC1D3B	ENSG00000274808		
KRT15	ENSG00000171346		
KRT19	ENSG00000171345		
KRT14	ENSG00000186847		
STAT3	ENSG00000168610		
SOCS3	ENSG00000184557		
DSC3	ENSG00000134762		
EFCAB8	ENSG00000215529		
TNNC2	ENSG00000101470		
FXYD7	ENSG00000221946		
CXCL17	ENSG00000189377		
CEACAM1	ENSG00000079385		
IGSF23	ENSG00000216588		
SLC5A4	ENSG00000100191		

A

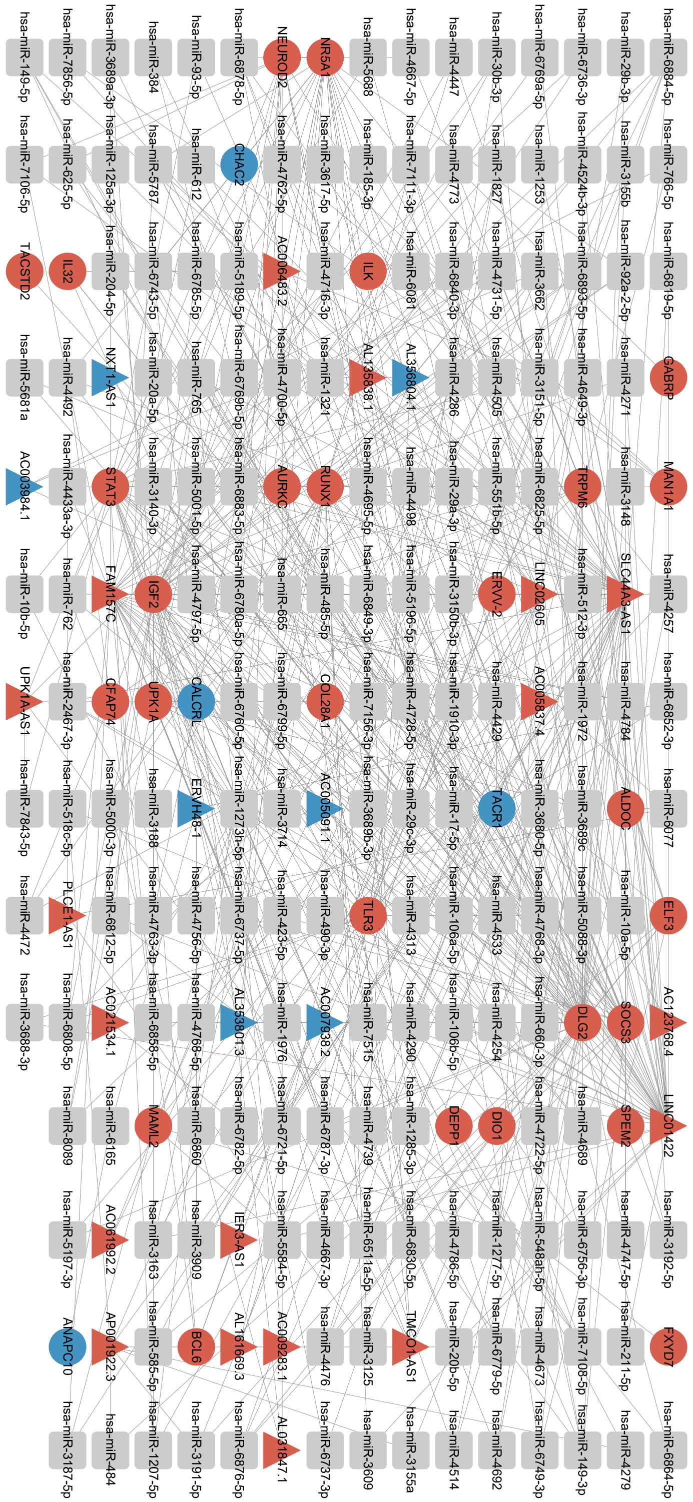

hsa-miR-149-5p
hsa-miR-7106-5p
TACSTD2
hsa-miR-5681a
AC003984.1
hsa-miR-10b-5p
UPK1A-AS1
hsa-miR-7843-5p
hsa-miR-4472
hsa-miR-3688-3p
hsa-miR-7856-5p
hsa-miR-625-5p
IL32
hsa-miR-4492
hsa-miR-4433a-3p
hsa-miR-762
hsa-miR-2467-3p
hsa-miR-518c-5p
PLCE1-AS1
hsa-miR-6808-5p
hsa-miR-8089
hsa-miR-5197-3p
ANAPC10
hsa-miR-3187-5p
hsa-miR-3689a-3p
hsa-miR-125a-3p
hsa-miR-204-5p
NXT1-AS1
STAT3
FAM157C
CFAP74
hsa-miR-5000-3p
hsa-miR-6812-5p
AC021534.1
hsa-miR-6165
AC061992.2
AP001922.3
hsa-miR-484
hsa-miR-384
hsa-miR-5787
hsa-miR-6743-5p
hsa-miR-20a-5p
hsa-miR-3140-3p
IGF2
UPK1A
hsa-miR-3188
hsa-miR-4763-3p
hsa-miR-6858-5p
MAML2
hsa-miR-3163
hsa-miR-585-5p
hsa-miR-1207-5p
hsa-miR-93-5p
hsa-miR-612
hsa-miR-6785-5p
hsa-miR-765
hsa-miR-5001-5p
hsa-miR-4797-5p
CALCRL
ERVH48-1
hsa-miR-4756-5p
hsa-miR-4768-5p
hsa-miR-6860
hsa-miR-3909
BCL6
hsa-miR-3191-5p
hsa-miR-6878-5p
CHAC2
hsa-miR-5189-5p
hsa-miR-6769b-5p
hsa-miR-6883-5p
hsa-miR-6780a-5p
hsa-miR-6760-5p
hsa-miR-1273h-5p
hsa-miR-6737-5p
AL353801.3
hsa-miR-6792-5p
IER3-AS1
AL161669.3
hsa-miR-6876-5p
NEUROD2
hsa-miR-4762-5p
AC006483.2
hsa-miR-4700-5p
AURKC
hsa-miR-665
hsa-miR-6799-5p
hsa-miR-3714
hsa-miR-423-5p
hsa-miR-1976
hsa-miR-6721-5p
hsa-miR-5584-5p
AC009283.1
AL031847.1
NR5A1
hsa-miR-3617-5p
hsa-miR-4716-3p
hsa-miR-1321
RUNX1
hsa-miR-485-5p
COL28A1
AC005091.1
hsa-miR-490-3p
AC007938.2
hsa-miR-6787-3p
hsa-miR-4667-3p
hsa-miR-4476
hsa-miR-6737-3p
hsa-miR-5688
hsa-miR-185-3p
ILK
AL135938.1
hsa-miR-4695-5p
hsa-miR-6849-3p
hsa-miR-7156-3p
hsa-miR-3689b-3p
TLR3
hsa-miR-7515
hsa-miR-4739
hsa-miR-6511a-5p
hsa-miR-3125
hsa-miR-3609
hsa-miR-4667-5p
hsa-miR-7111-3p
hsa-miR-6081
AL356804.1
hsa-miR-4498
hsa-miR-5196-5p
hsa-miR-4728-5p
hsa-miR-29c-3p
hsa-miR-4313
hsa-miR-4290
hsa-miR-1285-3p
hsa-miR-6830-5p
TMCO1-AS1
hsa-miR-3155a
hsa-miR-4447
hsa-miR-4773
hsa-miR-6840-3p
hsa-miR-4286
hsa-miR-29a-3p
hsa-miR-3150b-3p
hsa-miR-1910-3p
hsa-miR-17-5p
hsa-miR-106a-5p
hsa-miR-106b-5p
DEPP1
hsa-miR-4786-5p
hsa-miR-20b-5p
hsa-miR-4514
hsa-miR-30b-3p
hsa-miR-1827
hsa-miR-4731-5p
hsa-miR-4505
hsa-miR-551b-5p
ERVV-2
hsa-miR-4429
TACR1
hsa-miR-4533
hsa-miR-4254
DIO1
hsa-miR-1277-5p
hsa-miR-6779-5p
hsa-miR-4692
hsa-miR-6769a-5p
hsa-miR-1253
hsa-miR-3662
hsa-miR-3151-5p
hsa-miR-6825-5p
LINC02605
AC068337.4
hsa-miR-3680-3p
hsa-miR-4768-3p
hsa-miR-660-3p
hsa-miR-4722-5p
hsa-miR-548ah-5p
hsa-miR-4673
hsa-miR-6749-3p
hsa-miR-6736-3p
hsa-miR-4524b-3p
hsa-miR-6893-5p
hsa-miR-4649-3p
TRPM6
hsa-miR-512-3p
hsa-miR-1972
hsa-miR-3689c
hsa-miR-5088-3p
DLG2
hsa-miR-4689
hsa-miR-6756-3p
hsa-miR-7108-5p
hsa-miR-149-3p
hsa-miR-29b-3p
hsa-miR-3155b
hsa-miR-92a-2-5p
hsa-miR-4271
hsa-miR-3148
SLC44A3-AS1
hsa-miR-4784
ALDOC
hsa-miR-10a-5p
SOCS3
SPEM2
hsa-miR-4747-5p
hsa-miR-211-5p
hsa-miR-4279
hsa-miR-6884-5p
hsa-miR-766-5p
hsa-miR-6819-5p
GABRP
MAN1A1
hsa-miR-4257
hsa-miR-6852-3p
hsa-miR-6077
ELF3
AC123768.4
LINC01422
hsa-miR-3192-5p
FXYD7
hsa-miR-6864-5p

B

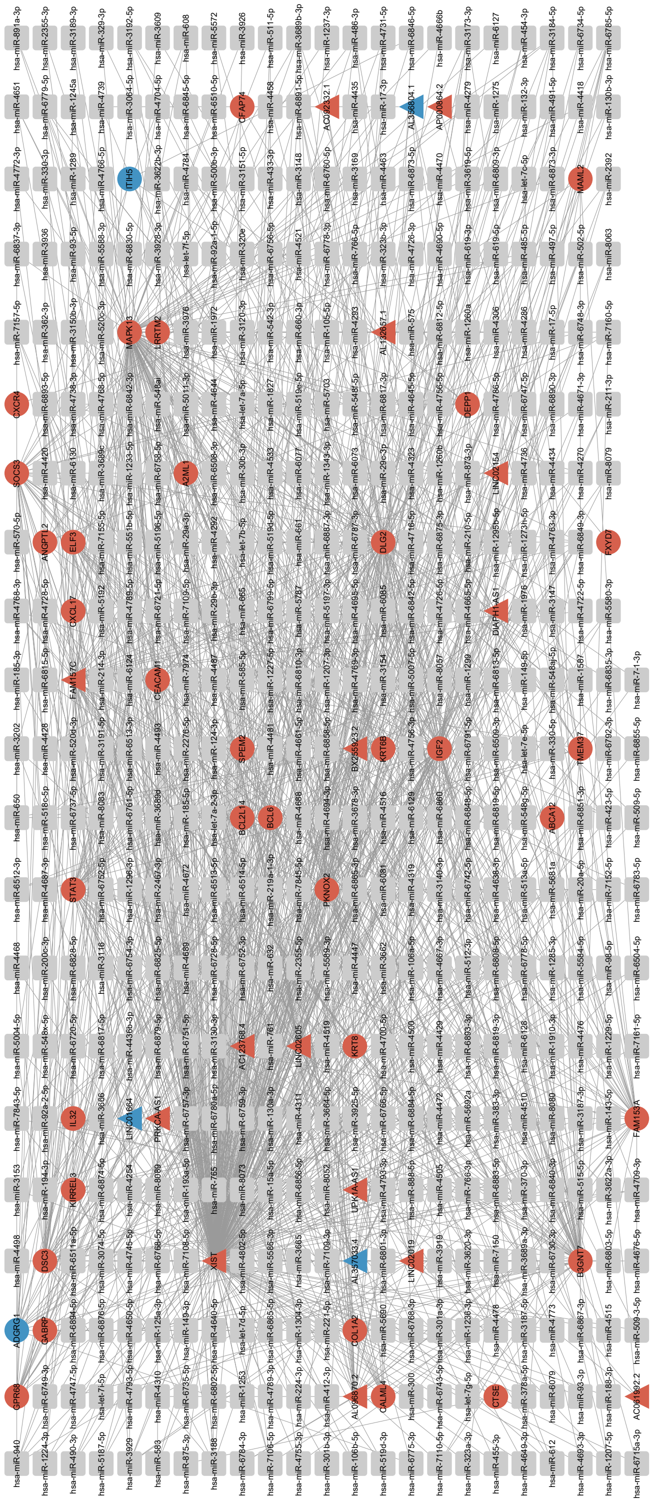

Supplementary Figure S1. full-sized Figure 8: mRNA-miRNA-lncRNA ceRNA networks between SC/DMS114-conditioned group and control group (**A**), SC-conditioned group and control group (**B**) .
